# Age–Period–Cohort Analysis of Trends in Infectious Disease Mortality in South Korea from 1983 to 2017

**DOI:** 10.3390/ijerph18030906

**Published:** 2021-01-21

**Authors:** Hee Sook Kim, Sang Jun Eun

**Affiliations:** 1 Central Disease Control Headquarters, Korea Disease Control and Prevention Agency, Cheongju 28159, Korea; nikita1025@korea.kr; 2 Department of Preventive Medicine, Chungnam National University College of Medicine, Daejeon 35015, Korea

**Keywords:** mortality, communicable diseases, age–period–cohort analysis, trends, Republic of Korea

## Abstract

We aimed to describe the infectious disease (ID) mortality trends and evaluate age–period–cohort (APC) effects on ID mortality in Korea. Using cause-of-death and census population estimates data from 1983–2017, age-standardized ID mortality trends were investigated by joinpoint regression analysis. The APC effects on ID mortality were estimated using intrinsic estimator models. The age effect showed a J-shaped concave upward curve. Old age, especially ≥70 years, was a critical factor for ID deaths. Similar to the W-shaped period curve, ID mortality rapidly decreased due to economic development and the expansion of health coverage in the 1980s, decelerated with increasing inequality, surged due to the 1997 economic crisis, and has gradually increased since the mid-2000s. The cohort effect showed an inverted U-shape. The increasing cohort effect due to the deterioration of living standards led to a decreasing trend after the independence of Korea. Notwithstanding the slowdown during the 1950–1953 Korean War, educational expansion, economic growth, fertility reduction, and the improvement of ID-related policies might have led to a continued decline among the cohorts born since the 1960s. Diverse socioeconomic events may have influenced ID mortality trends in Korea via period and cohort effects. Policies to reduce the growing burden of ID deaths should be further improved.

## 1. Introduction

Infectious disease (ID) is a leading cause of death and premature death worldwide, despite a decreasing trend [1]. In 2017, nearly 9 million people died and more than 420 thousand years of life were lost due to IDs, accounting for 15% of all deaths and 25% of years of life lost, respectively [1]. Particularly, ID is the leading cause of death among children under 15 years of age, comprising almost half of the total deaths [1]. Although the burden of IDs is disproportionately concentrated in low- and middle-income countries [1], it remains a major public health threat in high-income countries [2,3].

In South Korea (hereafter referred to as Korea), ID mortality was on a decline until the mid-1990s; however, it has been continuously increasing since the mid-2000s after a temporary increase during the 1997 economic crisis [2]. An increase in mortality due to pneumonia and sepsis has led to a recent unfavorable trend [2,4]. In 2018, pneumonia was the third leading cause of death, while sepsis was the 9th and 10th leading cause of death in women and men, respectively [4]. These diseases, along with tuberculosis, continue to contribute to the socioeconomic gap in mortality and life expectancy [2,5].

Mortality trends can be affected by biological changes associated with aging, social impact at a particular time, and shared experiences among a specific generation [6]. Vulnerable age groups differed depending on the type of ID. Although the influenza mortality rate showed a trimodal age distribution; high in children, young adults, and the elderly [7], overall ID mortality was reported to increase incrementally with age [8]. Social changes over time, such as economic development and crisis [8,9,10], urbanization [11], and enhancement of healthcare and public health systems [3,8,9], might influence ID mortality. The risk of death from IDs tended to be lower among those born during periods with improved living conditions than those who were not [8,9]. Therefore, disentangling age, period, and cohort (APC) effects would help comprehend the possible reasons underlying ID mortality trends.

Research on ID mortality trends has primarily focused on temporal changes [2,3,8]. Several studies that examined the APC effects on ID mortality were focused only on specific IDs like influenza and pneumonia [7,9]. Research concerning the APC effects on overall ID mortality is scarce [8] and has never been undertaken in Korea. The findings of a previous study that analyzed the temporal trend of ID mortality in Korea were inappropriate for the interpretation of temporal changes and international comparisons because of the use of crude mortality rates [2]. Korea is among the world’s fastest aging [4] and most educated societies [12]. It has achieved rapid economic growth and suffered from economic crisis [13]. The Korean government has established various measures and policies for ID control and prevention, including legislations, the implementation of national immunization programs, and the expansion of health coverage [14,15]. The study aimed to describe ID mortality trends and estimate APC effects on ID mortality in Korea from 1983 to 2017, thereby exploring potential factors that might affect ID mortality trends.

## 2. Materials and Methods

### 2.1. Data Sources

Using data from the national death certificate and the population census of Statistics Korea between 1983 and 2017, the annual number of ID deaths and the corresponding mid-year census population estimates were obtained by sex and age [4]. The underlying causes of death in Korea were classified according to the International Classification of Diseases (ICD) 9th Revision until 1994 and have been coded using ICD-10 since 1995. For the number of deaths from IDs that were coded with ICD-9 from 1983 to 1994, Statistics Korea publicly provides the number of ID deaths changed into the ICD-10 codes using the World Health Organization ICD-9/ICD-10 translator [16]. Accordingly, ID deaths were defined by ICD-10 codes of A00–B99, G00, G03, G04, I00–I09, J09–J18, J86, and M86 [2], which were the codes available in aggregate death certificate data among the codes covering IDs [17]. A total of 440,154 ID deaths were included in the analysis, excluding 126 deaths from IDs from an unknown age group.

### 2.2. Statistical Analysis

Age-standardized mortality rates (ASMRs) from IDs per 100,000 person-years were calculated by the direct method, using the world standard population as the reference population [18], for the overall population, both sexes, and age groups (0–4, 5–24, 25–44, 45–64, and ≥65 years). Joinpoint regression analysis was used to identify significant changes in the slope of the ID mortality trend, using the Monte Carlo permutation method [19]. The estimated annual percent change (EAPC) was calculated for each linear trend to measure the pace of change in ASMR and the corresponding 95% confidence intervals (CIs) were computed [19].

To assess whether APC components affected ID mortality, data on ID deaths and mid-year populations were aggregated into 5-year age groups (0–4, 5–9, …, ≥80 years) and 5-year periods (1983–1987, 1988–1992, …, 2013–2017). Partially overlapping birth cohorts were derived (1903, 1908, …, 2013) by subtracting the mean age from the mean period, which represented nine birth-years (e.g., the 2013 cohort included those born between 2009 and 2017). To evaluate the independent effects of APC components while avoiding linear dependence (i.e., period = age + cohort), APC models estimated using the intrinsic estimator (IE), based on log-linear Poisson regression, were fitted with the “apc_ie” module in Stata [6]. The APC coefficient estimates from the IE models were exponentiated to produce rate ratios (RRs), which indicate the risk of ID deaths of a specific APC category relative to the average risk of all APC categories combined. The goodness-of-fit statistics suggested that a full APC model was preferable over any combination of APC components (Supplementary Table S1).

Analyses were performed using the Joinpoint Regression Program V.4.8.0.1 [19] and Stata V.15.1 (StataCorp., College Station, TX, USA). This study was exempted from ethical approval by the Chungnam National University Institutional Review Board (IRB No. 202005-SB-044-01).

## 3. Results

**Figure 1:**
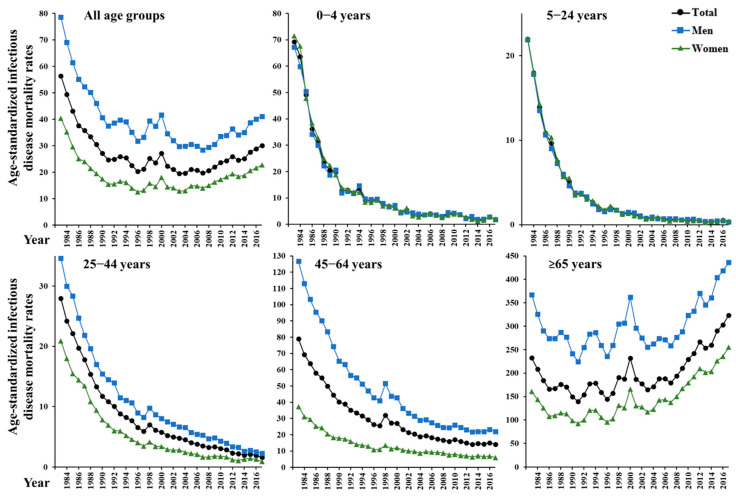
Trends in age-standardized mortality rates from infectious diseases by sex and age group (all age groups, 0–4 years, 5–24 years, 25–44 years, 45–64 years, and ≥65 years) in South Korea from 1983 to 2017.

Figure 1, Table 1, and Supplementary Table S2 present trends and changes in ASMRs due to IDs in Korea from 1983–2017. The findings of the joinpoint regression models are shown in Table 1. During the study period, the ASMR declined annually by 1.8%, plummeted from 56.4 in 1983 to 24.7 in 1990 (EAPC −10.1, 95% CI −12.3 to −7.7), then slowed down (EAPC −1.5, 95% CI −2.3 to −0.7) with a temporary rise in the late 1990s, and has gradually increased since around the mid-2000s (EAPC 4.0, 95% CI 2.8 to 5.2). The ASMRs by age group exhibited concave upward trends with a steep to moderate negative slope in the <65 age groups. However, the elderly group has had a positive slope since the mid-2000s and was the only group with no ID mortality reduction (EAPC 1.4, 95% CI −0.7 to 3.5). There were upward inflections around 1998 in the ≥25 age groups, which were more pronounced among the ≥65 age group and men. Sex differences in ASMRs by age groups were variable but minimal in the 0–24 age groups, whereas in the ≥25 age groups, ASMRs in women were consistently lower than in men or the entire population.

**Figure 2:**
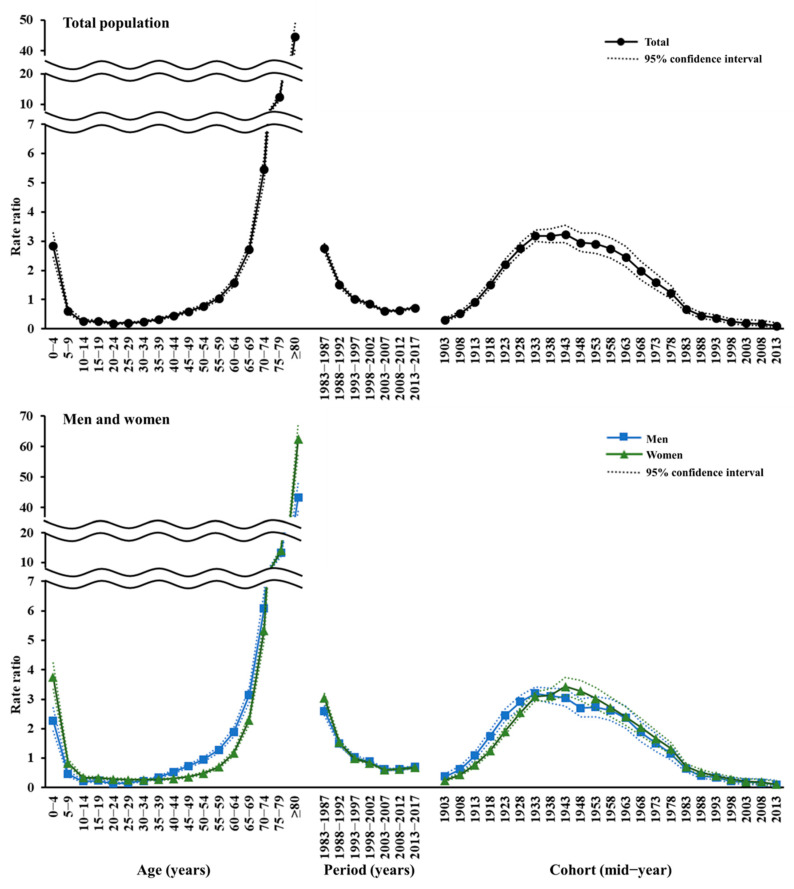
Age, period, and cohort effects on infectious disease mortality in South Korea from 1983 to 2017.

The findings of the IE models in Figure 2 and Supplementary Table S3 suggested that each of the APC components substantially influenced ID mortality. The age effect showed a J-shaped concave upward curve with the lowest mortality in the 20–24 age group (RR 0.18, 95% CI 0.16 to 0.22), a surge in the 70–74 age group (RR 5.46, 95% CI 5.04 to 5.91), and the highest in the ≥80 age group (RR 44.68, 95% CI 40.75 to 48.98). The age effect gradually increased in men from 30s to 50s, but did not change significantly in women from 15 to 49 years. The period effect trend followed the concave upward tendency of the joinpoint regression analysis results, with a downward inflection after 1998–2002, with the lowest in 2003–2007 (RR 0.61, 95% CI 0.58 to 0.64). The cohort effect increased in those born from 1903 to 1943, gradually declined from the 1948 cohort after peaking for the 1933–1943 cohorts (RR 3.24, 95% CI 2.96 to 3.54 for the 1943 cohort), rapidly decreased from the 1963 cohort, and then slowed down from the 1988 cohort. The risk of ID deaths in men peaked among the 1933 cohort (RR 3.20, 95% CI 2.99 to 3.42), earlier than women (RR 3.43, 95% CI 3.15 to 3.73 for the 1943 cohort), and inflected upward in the 1953 cohort.

## 4. Discussion

To examine factors that may have potentially affected ID mortality changes in Korea from 1983–2017, we analyzed the ID mortality trends and evaluated the effects of APC components on ID mortality. The ID mortality trend showed a W-shaped curve with an upward inflection in the late 1990s. APC components independently influenced this trend significantly.

The J-shaped age effects reflected changes in the immune response to IDs over a lifetime, as the immune system is relatively immature at birth, matures with growth and development, and then declines in function in the elderly [20]. Old age, especially ≥70 years, was a critical factor for ID deaths in Korea, which could be attributed to biological susceptibility to IDs, but might also be ascribed to the socioeconomic vulnerability of the elderly. For instance, Korea’s relative poverty rate (proportion of population with incomes below 50% of the median income) for the elderly has been around 45%, over three times higher than the OECD and national averages [21]. Among the elderly, the rate was even higher among those who were older, lived alone, female, and less educated [21]. In 2017, 58.4% of the elderly were undereducated (no education to primary education), and 30.9% were working mostly in precarious employment, including self-employment, to make a living [4]. The socially disadvantaged status of the elderly in Korea might have contributed to a high risk of ID deaths [2,22,23].

The ASMRs in men were higher than in women among the ≥25 age groups and little change in age effect was observed among women of reproductive age. These findings could be explained by behavioral (e.g., unhealthy behaviors of men such as smoking and alcohol intake) or biological (e.g., regulatory role of the X chromosome for immune functioning, immunosuppressive effects of male sex hormones, and immunoprotective effects of female sex hormones) differences between the sexes [24].

The ASMR from IDs in Korea had declined until the mid-1990s, increased temporarily during the 1997 economic crisis, and then gradually increased. Although it was difficult to directly compare the ID mortality trends due to differences in the definition and epidemiology of IDs and the standard population, the ID mortality rates remained unchanged in the United States from 1980–2014 [25], declined overall from 1980–2011 in Spain [17], and decreased from 1958 to the mid-1990s, increased from 1997–2003, and then decreased in Thailand [26]. In these countries, changes in mortality due to the epidemics of HIV/AIDS were a main factor affecting the ID mortality trends [17,25,26]. On the contrary, in Korea, HIV/AIDS mortality has been low [27], and changes in tuberculosis and pneumonia mortalities played a major role in the trend in ID mortality [2,4].

The dramatic drop in period effects in the 1980s could be primarily attributed to rapid economic growth, which meant an increase in available resources for individuals, improved public health measures, and significant progress towards universal health coverage [2,8,9,11]. Previous research reported that the reduction of ID deaths in the 1980s was driven by decreases in infant and tuberculosis-related deaths [2]. Implementation of the free mandatory immunization program for children in 1974 and better sanitation and hygiene alongside urbanization might have contributed to the reduction of infant ID mortality by decreasing mortality rates from vaccine-preventable and intestinal infections [4,11,15]. Increased access to healthcare by expanding health coverage (29.6% in 1980 to the entire population in 1989) might have made a substantial contribution to reducing ID mortality risk, including tuberculosis, pneumonia, and infant ID mortality [2,15]. The decline in tuberculosis mortality, the leading cause of ID deaths until the 1990s, could also be attributed to the enhanced control of tuberculosis, such as vaccination, early detection, and effective treatment (e.g., use of rifampicin in treatment regimens from 1980, which increased the recovery rate by reducing the duration of anti-tuberculosis treatment by 50%) [2,15].

The slowdown in the ASMR decline in the early 1990s might be linked to changes in socioeconomic inequality. Korea’s economic growth until the 1980s led to improvements in both the average income and income distribution. However, globalization and economic restructuring led to a rise in income inequality from the early 1990s [28,29]. Growing socioeconomic inequality may negatively influence ID mortality by increasing the risk of exposure to IDs (e.g., overcrowding and unhygienic environment), susceptibility to IDs (e.g., reduced immunity due to high stress levels and poor nutritional status), and disease severity of IDs (e.g., decreased access to quality healthcare and unhealthy behaviors) [10].

The 1997 economic crisis (December 1997 to August 2001) had devastating consequences for the socioeconomic contexts in Korea. Comprehensive structural re-adjustments and the subsequent neoliberal economic policies strikingly increased labor market flexibility, layoffs, job insecurity, and precarious employment, thereby increasing income inequality [13,28,30]. The adverse effects of the economic crisis on ID mortality were more prominent in the middle-aged male economically active population and the elderly, probably due to massive layoffs of workers in their 40s to 50s, low female labor-force participation rate, and a large proportion of older people with low socioeconomic status [4,30]. Although ID mortalities among the <65 age groups have decreased slowly since the early 1990s, they are still affected by the economic crisis in the economically active age groups. However, ID mortalities have been steadily decreasing until recently, consistent with previous findings that the economic crisis may not have long-term effects on ID mortality [10]. The findings may be attributed to improved health and hygiene awareness and continued advances in healthcare and public health measures [15]. However, given the limited social safety net in Korea, the elderly suffered the most due to the economic crisis, with little social protection [2,13]. Notwithstanding the Korean government’s efforts to expand and reinforce the social safety net in response to the economic crisis, they were still insufficient to ameliorate socioeconomic disadvantages of the elderly [30], thereby increasing ID mortality risk during the economic crisis and thereafter [23]. Moreover, Korea became an aging society with a 7.2% share of the elderly population in 2000, and an aged society with 14.3% in 2018 [4]. Combined with an epidemiological transition to non-communicable diseases and increasing socioeconomic inequality [23], population aging has led to an increase in elderly people with immunocompromising conditions [2]. The proportion of older people with multimorbidity (having three or more chronic conditions) increased from 30.7% in 2008 to 51.0% in 2017 [4]. An increasing proportion of elderly people with multimorbidity might have increased vulnerability to IDs, possibly raising the ASMR of the elderly [22]. On the other hand, a rise in nosocomial infections and antimicrobial resistance from the 1990s might have contributed to increasing ASMRs from IDs such as pneumonia and sepsis, especially in the elderly [2,15]. As Korea has the world’s fastest aging population [4], there is a possibility that the burden of IDs in the elderly will persistently increase [31]. Therefore, policies to mitigate ID mortality in the elderly should be strengthened, including social protection policies (e.g., public income transfers), and should not be limited to healthcare policies [22,23].

The cohort effect showed an inverted U-shape. The increasing RR of ID deaths among the cohorts born between 1903 and 1943 might be the consequences of deteriorating living standards of the Korean people during the 1910–1945 Japanese forced occupation period [29]. It has been noted that low socioeconomic circumstances, poor nutritional conditions, and exposure to IDs in early life may be associated with an increase in ID mortality in later life [32,33]. Throughout this period, Koreans experienced worsening nutritional status, decreased average height, lack of educational opportunities, widening socioeconomic inequality, and increasing ID mortality [14,29,34], possibly resulting in an increased ID mortality risk. Extreme exploitation of material and human resources by Japan to secure war supplies for the China–Japan War and World War II between 1931 and 1945 exacerbated the living standards for the Koreans [29], which might have caused the peak of ID mortality risk in the 1933–1943 cohorts. Among the 1933–1943 cohorts, the RR slightly decreased in men after peaking in the 1933 cohort, but gradually increased in women, peaking in the 1943 cohort. Although the primary education enrolment rate increased in the mid-1930s, widespread sex disparities meant the rate was 60% for males and 20% for females in 1940 [35]. Sex disparities in educational experiences might have contributed to the early emergence of the peak in men compared to women [22].

After the independence in 1945, extreme socioeconomic inequality was rapidly alleviated by the land reform that lowered the high percentage rent rate and brought the Japanese-owned farmland back to Korean farmers [29]. The release of suppressed educational demand with the full implementation of compulsory primary education in 1954 led to an increase in enrolment rates for primary education [34], probably reducing the RR in the 1948 cohort. The absence of any significant reduction in RR in the 1953 cohort could be attributed to the devastating influence of the 1950–1953 Korean War, consistent with previous findings that children born during the war had an elevated mortality risk at older ages than adjacent cohorts [36,37]. The war created a disrupted social system, further leading to a poor nutritional status, inadequate sanitation, overcrowded living conditions, and impaired access to healthcare and public health services, leading to ID epidemics such as smallpox, typhus, and tuberculosis [15,22]. These detrimental war-related experiences in the 1953 cohort might have increased the risk of ID deaths [33,37]. However, the Korean War seemed to have affected only the male ID mortality risk, which might be ascribed to a higher vulnerability to IDs (e.g., tuberculosis) in men than women [24], selective survival bias due to the lack of parental investment in unhealthy female children, and greater effects of husband’s socioeconomic status on women’s health in later life rather than the birth year of women [37].

The steep decline in RR among the cohorts born in the 1960s to the early 1980s could be attributed to rapid socioeconomic developments since the 1960s [8,9,15]. The economy grew swiftly with an annual average rate of nearly 10%, restoring the infrastructure damaged by the war in the 1950s, while keeping the level of inequality relatively low [28]. The gross national income per capita increased about 18-fold from USD 120 in 1962 to USD 2150 in 1983 [4]. The primary education enrolment rate had already reached 96% in 1959 and the consistent increase in the enrolment rate and the advancement rate to secondary or higher education made Korea one of the world’s most educated societies in the late 1990s [12,34]. Additionally, the government implemented various programs and policies to control and prevent IDs, including the enactment and amendment of ID-related legislations, the implementation of a parasite eradication program, the expansion of a national immunization program, and the establishment of an ID management system [14,15]. The accumulation of physical and human capital and the government’s efforts might have contributed to the reduction of RR in the 1963–1983 cohorts [22].

Although ID-related programs and policies had been strengthened in the 1980s and afterwards [14,15], the RR decline slowed down from the 1988 cohort. Similarly, the total fertility rate in Korea steeply decreased from 4.53 in 1970 to 1.74 in 1984, maintaining this level until the late 1990s, and fell to 1.18 in 2002, the lowest-low fertility level, and has continues to decline to the present [4]. Previous studies have shown that the reduction in fertility rate is associated with a decrease in childhood ID mortality, due to fewer opportunities for transmission and more parental care for each offspring [31,38], which might partly explain the decreasing trend of RR.

Several limitations should be noted. First, the cause-of-death data in Korea do not have a very high proportion of deaths registered to a well-defined cause (75.8% from 1985–2016) [39]. The classification accuracy of ID deaths was reported to be 66.7% [40]. The misclassification bias might have underestimated the ID mortality. Second, since only aggregate data on ID deaths were available, some IDs could not be included in the analysis, such as perinatal infections (P23, P35–39), cardiac infections (I30, I33), and genitourinary infections (N10–13) [2]. Third, it was not possible to trace the trajectory of ID mortality over the whole life course of a particular cohort. Fourth, the findings of this study do not demonstrate any association or causation between ID mortality and a specific policy or socioeconomic event, but might have implications for inferring potential reasons for ID mortality changes. Fifth, the classification accuracy of the cause of death may have changed over time due to advances in diagnostic technology, which might make it difficult to compare ID mortalities between time periods [41]. Moreover, changes in the ICD coding scheme may have affected the ID mortality trend, therefore, the trend might reflect a change in the cause-of-death coding scheme rather than a change in the actual ID mortality [41]. Lastly, despite discussing potential drivers for ID mortality changes using available evidence, there might be other explanations for these changes. Further research is needed to better understand ID mortality trends.

## 5. Conclusions

The ID mortality trends in Korea from 1983 to 2017 seem to have been affected by various socioeconomic events, including educational expansion, socioeconomic inequality changes, economic growth and crisis, demographic transition, the Korean War, enhanced access to healthcare, and the improvement of ID control and prevention measures, via period and cohort effects. In difficult times, such as the economic crisis or deepening socioeconomic inequality, a vulnerable population with low education or income might be more at risk of dying from IDs. However, the expansion of education and health coverage and strengthening public health might have had an impact on mitigating these risks at the population level. The recent increasing ID mortality trend suggests an urgent need to protect elderly people from the negative consequences of IDs. Alongside healthcare and public health policies, social protection policies should be further improved to reduce the growing burden of ID deaths.

## Figures and Tables

**Table 1:** Changes in age-standardized mortality rates from infectious diseases by sex and age group in South Korea from 1983 to 2017.

Age Group	Total			Men			Women		
	Periods	EAPC (95% CI)	*p*-Value for Slope Change	Periods	EAPC (95% CI)	*p*-Value for Slope Change	Periods	EAPC (95% CI)	*p*-Value for Slope Change
All ages	1983–1990	−10.1 (−12.3, −7.7)	-	1983–1990	−8.4 (−10.2, −6.6)	-	1983–1990	−11.2 (−13.0, −9.4)	-
	1990–2007	−1.5 (−2.3, −0.7)	<0.0001	1990–1996	−3.1 (−6.6, 0.5)	0.0114	1990–1997	−3.2 (−6.3, −0.0)	0.0001
				1996–2000	4.4 (−3.1, 12.5)	0.0752	1997–2000	8.1 (−10.6, 30.7)	0.2457
				2000–2003	−9.7 (−21.4, 3.8)	0.0696	2000–2003	−9.0 (−24.5, 9.7)	0.1943
				2003–2007	−0.4 (−7.3, 6.9)	0.2070	2003–2017	4.1 (3.5, 4.7)	0.1512
	2007–2017	4.0 (2.8, 5.2)	<0.0001	2007–2017	3.5 (2.5, 4.4)	0.2756			
	1983–2017	−1.8 (−2.5, −1.1)		1983–2017	−1.8 (−3.5, −0.0)		1983–2017	−1.6 (−3.9, 0.7)	
0–4 years	1983–1989	−20.0 (−21.8, −18.1)	-	1983–1989	−20.0 (−22.1, −17.8)	-	1983–1991	−18.6 (−19.9, −17.3)	-
	1989–2003	−10.2 (−11.5, −8.9)	<0.0001	1989–2004	−9.5 (−10.9, −8.1)	<0.0001	1991–2004	−9.4 (−11.1, −7.6)	<0.0001
	2003–2017	−4.8 (−7.4, −2.1)	0.0008	2004–2017	−4.5 (−7.9, −0.9)	0.0098	2004–2017	−4.9 (−8.1, −1.7)	0.0175
	1983–2017	−9.9 (−11.0, −8.7)		1983–2017	−9.6 (−11.0, −8.2)		1983–2017	−10.0 (−11.3, −8.7)	
5–24 years	1983–1991	−19.5 (−20.4, −18.6)	-	1983–1986	−21.7 (−25.2, −18.1)	-	1983–1991	−19.3 (−20.3, −18.3)	-
	1991–2004	−11.6 (−13.0, −10.2)	<0.0001	1986–1995	−17.0 (−18.6, −15.4)	0.0234	1991–2007	−11.9 (−13.0, −10.8)	<0.0001
	2004–2017	−5.5 (−8.0, −2.9)	0.0001	1995–2017	−7.7 (−8.8, −6.6)	<0.0001	2007–2017	−2.7 (−7.1, 1.9)	0.0002
	1983–2017	−11.3 (−12.3, −10.3)		1983–2017	−11.6 (−12.4, −10.7)		1983–2017	−11.1 (−12.4, −9.9)	
25–44 years	1983–1996	−10.6 (−11.0, −10.2)	-	1983–1989	−10.9 (−11.6, −10.1)	-	1983–1995	−12.1 (−13.0, −11.2)	-
				1989–1996	−8.7 (−9.7, −7.7)	0.0013			
	1996–1999	−0.6 (−12.9, 13.4)	0.1121	1996–1999	−1.8 (−11.3, 8.8)	0.1542	1995–2017	−6.4 (−7.1, −5.6)	<0.0001
	1999–2017	−6.9 (−7.3, −6.5)	0.3174	1999–2010	−6.3 (−6.9, −5.7)	0.3480			
				2010–2017	−9.1 (−10.6, −7.7)	0.0011			
	1983–2017	−7.8 (−8.9, −6.7)		1983–2017	−7.8 (−8.7, −6.9)		1983–2017	−8.4 (−9.0, −7.9)	
45–64 years	1983–1990	−8.6 (−9.2, −8.0)	-	1983–1996	−7.9 (−8.3, −7.5)	-	1983–1993	−8.8 (−9.8, −7.8)	-
	1990–1996	−6.9 (−8.1, −5.6)	0.0196						
	1996–1999	3.3 (−5.7, 13.2)	0.0288	1996–1999	3.2 (−10.3, 18.7)	0.1084	1993–2017	−3.2 (−3.6, −2.9)	<0.0001
	1999–2002	−10.9 (−18.9, −2.2)	0.0278	1999–2004	−9.7 (−12.8, −6.4)	0.0694			
	2002–2013	−3.3 (−3.7, −2.8)	0.0802	2004–2017	−2.1 (−2.6, −1.6)	0.0001			
	2013–2017	0.1 (−2.1, 2.3)	0.0051						
	1983–2017	−4.8 (−5.9, −3.7)		1983–2017	−5.0 (−6.2, −3.8)		1983–2017	−4.9 (−5.3, −4.5)	
≥65 years	1983–1996	−2.3 (−3.9, −0.7)	-	1983–1996	−2.1 (−3.7, −0.5)	-	1983–1996	−2.5 (−4.2, −0.7)	-
	1996–2000	10.6 (−0.8, 23.4)	0.0290	1996–2000	9.8 (−0.7, 21.4)	0.0283	1996–2000	12.1 (−1.6, 27.8)	0.0386
	2000–2003	−10.3 (−25.2, 7.7)	0.0533	2000–2003	−12.1 (−25.7, 3.9)	0.0270	2000–2003	−8.1 (−25.9, 13.9)	0.1152
	2003–2017	5.1 (4.3, 5.9)	0.0869	2003–2017	4.3 (3.5, 5.1)	0.0453	2003–2017	5.5 (4.7, 6.3)	0.1974
	1983–2017	1.4 (−0.7, 3.5)		1983–2017	0.9 (−1.0, 2.8)		1983–2017	1.9 (−0.6, 4.4)	

Abbreviations: EAPC, estimated annual percent change; CI, confidence interval.

## Data Availability

Publicly available datasets were analyzed in this study. This data can be found here: http://kosis.kr/index/index.do.

## Supplementary Materials

The following are available online at https://www.mdpi.com/1660-4601/18/3/906/s1, Table S1: The goodness-of-fit statistics for the combination of age, period, and cohort components. Table S2: Age-standardized mortality rates from infectious diseases by sex and age group in South Korea from 1983 to 2017. Table S3: Rate ratios based on intrinsic estimator coefficients for age, period, and cohort for infectious disease deaths in South Korea from 1983 to 2017.

## References

[1] GBD 2017 Causes of Death Collaborators (2018). Global, regional, and national age-sex-specific mortality for 282 causes of death in 195 countries and territories, 1980-2017: A systematic analysis for the Global Burden of Disease Study 2017. Lancet.

[2] Choe Y.J., Choe S.A., Cho S.I. (2018). Trends in infectious disease mortality, South Korea, 1983-2015. Emerg. Infect. Dis..

[3] Armstrong G.L., Conn L.A., Pinner R.W. (1999). Trends in infectious disease mortality in the United States during the 20th century. JAMA.

[4] Statistics Korea Korean Statistical Information Service. http://kosis.kr/index/index.do.

[5] Jung-Choi K., Khang Y.H., Cho H.J., Yun S.C. (2014). Decomposition of educational differences in life expectancy by age and causes of death among South Korean adults. BMC Public Health.

[6] Yang Y., Fu W.J., Land K.C. (2004). A methodological comparison of age-period-cohort models: The intrinsic estimator and conventional generalized linear models. Sociol. Methodol..

[7] Oei W., Nishiura H. (2012). The relationship between tuberculosis and influenza death during the influenza (H1N1) pandemic from 1918-19. Comput. Math. Methods Med..

[8] Li Z., Wang P., Gao G., Xu C., Chen X. (2016). Age-period-cohort analysis of infectious disease mortality in urban-rural China, 1990-2010. Int. J. Equity Health.

[9] Wong I.O., Cowling B.J., Leung G.M., Schooling C.M. (2012). Trends in mortality from septicaemia and pneumonia with economic development: An age-period-cohort analysis. PLoS ONE.

[10] Suhrcke M., Stuckler D., Suk J.E., Desai M., Senek M., McKee M., Tsolova S., Basu S., Abubakar I., Hunter P. (2011). The impact of economic crises on communicable disease transmission and control: A systematic review of the evidence. PLoS ONE.

[11] Alirol E., Getaz L., Stoll B., Chappuis F., Loutan L. (2011). Urbanisation and infectious diseases in a globalised world. Lancet Infect. Dis..

[12] Sandefur G.D., Park H. (2007). Educational expansion and changes in occupational returns to education in Korea. Res. Soc. Strat. Mobil..

[13] Khang Y.H., Lynch J.W., Kaplan G.A. (2005). Impact of economic crisis on cause-specific mortality in South Korea. Int. J. Epidemiol..

[14] Chun B.C. (2011). Public policy and laws on infectious disease control in Korea: Past, present and prospective. Infect. Chemother..

[15] Korean Society of Infectious Diseases (2018). Korean History of Infectious Diseases II.

[16] World Health Organization International Classification of Diseases Translator: Ninth and Tenth Revisions: User’s Guide to Electronic Tables [Computer File]. http://www.who.int/iris/handle/10665/63974.

[17] Lopez-Cuadrado T., Llacer A., Palmera-Suarez R., Gomez-Barroso D., Savulescu C., Gonzalez-Yuste P., Fernandez-Cuenca R. (2014). Trends in Infectious Disease Mortality Rates, Spain, 1980–2011. Emerg. Infect. Dis..

[18] World Health Organization World Standard Population. https://apps.who.int/healthinfo/statistics/mortality/whodpms/definitions/pop.htm.

[19] National Cancer Institute Joinpoint Regression Program, Version 4.8.0.1. https://surveillance.cancer.gov/joinpoint/.

[20] Simon A.K., Hollander G.A., McMichael A. (2015). Evolution of the immune system in humans from infancy to old age. Proc. Biol. Sci..

[21] Ku I., Kim C.O. (2020). Decomposition analyses of the trend in poverty among older adults: The case of South Korea. J. Gerontol. B Psychol. Sci. Soc. Sci..

[22] Semenza J.C., Lindgren E., Balkanyi L., Espinosa L., Almqvist M.S., Penttinen P., Rocklöv J. (2016). Determinants and drivers of infectious disease threat events in Europe. Emerg. Infect. Dis..

[23] Bambra C., Riordan R., Ford J., Matthews F. (2020). The COVID-19 pandemic and health inequalities. J. Epidemiol. Community Health.

[24] Klein S.L., Flanagan K.L. (2016). Sex differences in immune responses. Nat. Rev. Immunol..

[25] Hansen V., Oren E., Dennis L.K., Brown H.E. (2016). Infectious disease mortality trends in the United States, 1980-2014. JAMA.

[26] Aungkulanon S., McCarron M., Lertiendumrong J., Olsen S.J., Bundhamcharoen K. (2012). Infectious disease mortality rates, Thailand, 1958–2009. Emerg. Infect. Dis..

[27] OECD (2019). HIV/AIDS.

[28] Shin K.-Y., Kong J. (2014). Why does inequality in South Korea continue to rise?. Korean J. Sociol..

[29] Jeong T. (2017). Korean living standards under Japanese colonial rule: A critical review of the longitudinal trajectory of stature. Rev. Korean Stud..

[30] Son M., Cho Y., Oh J., Kawachi I., Yi J., Kwon S. (2012). Social inequalities in life expectancy and mortality during the transition period of economic crisis (1993-2010) in Korea. Int. J. Equity Health.

[31] Williams J.R., Manfredi P. (2004). Ageing populations and childhood infections: The potential impact on epidemic patterns and morbidity. Int. J. Epidemiol..

[32] Galobardes B., Lynch J.W., Davey Smith G. (2004). Childhood socioeconomic circumstances and cause-specific mortality in adulthood: Systematic review and interpretation. Epidemiol. Rev..

[33] Bengtsson T., Lindström M. (2003). Airborne infectious diseases during infancy and mortality in later life in southern Sweden, 1766–1894. Int. J. Epidemiol..

[34] Heo S. (2012). Korea‘s liberation in 1945 and its economic development. J. Korean Indep. Mov. Stud..

[35] Oh S.-C., Kim K.-S. (1998). The increase of educational opportunity in Korea under the Japanese occupation: For whom the bell told?. SNU J. Educ. Res..

[36] Elo I.T., Preston S.H. (1992). Effects of early-life conditions on adult mortality: A review. Popul. Index.

[37] Lee C. (2014). In utero exposure to the Korean War and its long-term effects on socioeconomic and health outcomes. J. Health Econ..

[38] Reves R. (1985). Declining fertility in England and Wales as a major cause of the twentieth century decline in mortality. The role of changing family size and age structure in infectious disease mortality in infancy. Am. J. Epidemiol..

[39] GBD 2016 Causes of Death Collaborators (2017). Global, regional, and national age-sex specific mortality for 264 causes of death, 1980-2016: A systematic analysis for the Global Burden of Disease Study 2016. Lancet.

[40] Chung E.K., Shin H.Y., Shin J.H., Nam H.S., Ryu S.Y., Im J.S., Rhee J.A. (2002). Accuracy of the registered cause of death in a county and its related factors. Korean J. Prev. Med..

[41] Lim D., Ha M., Song I. (2014). Trends in the leading causes of death in Korea, 1983-2012. J. Korean Med. Sci..

