# Supplementary material for: Age–Period–Cohort Analysis of Trends in Infectious Disease Mortality in South Korea from 1983 to 2017
Source: Int J Environ Res Public Health. 2021 Jan 21;18(3):906. doi: 10.3390/ijerph18030906 (PMC7908575; doi:10.3390/ijerph18030906)
Supplement: Supplementary file 1 [file ijerph-18-00906-s001.pdf]

Supplementary Table S1 The goodness-of-fit statistics for the combination of age, period and cohort components

Model	Total				Men				Women			
	d.f.	Log-likelihood	Deviance	AIC	d.f.	Log-likelihood	Deviance	AIC	d.f.	Log-likelihood	Deviance	AIC
Age	102	-57895.3	1124.5	973.3	102	-33101.1	638.9	556.6	102	-26024.6	500.8	437.7
Period	112	-617324.2	11013.9	10375.3	112	-343949.9	6132.7	5780.8	112	-302096.7	5385.9	5077.4
Cohort	96	-178328.2	3703.8	2997.5	96	-82107.7	1699.8	1380.3	96	-110873.2	2299.8	1863.8
Age and period	96	-38430.8	789.3	646.3	96	-18179.2	367.9	305.9	96	-18184.0	368.8	306.0
Age and cohort	80	-10720.5	254.4	180.8	80	-6337.2	145.5	107.2	80	-4150.6	91.7	70.4
Period and cohort	90	-92956.3	2053.6	1562.8	90	-42077.2	923.5	707.7	90	-58918.1	1298.5	990.7
Age, period and cohort	75	-1789.9	33.2	30.8	75	-1407.6	23.7	24.4	75	-851.5	9.8	15.0

d.f., degree of freedom; AIC, Akaike information criterion

Supplementary Table S2 Age-standardized mortality rates from infectious diseases by sex and age group in South Korea from 1983 to 2017

Sex	Year	All age			0-4 years			5-24 years			25-44 years			45-64 years			≥65 years		
		ASMR	95% confidence interval		ASMR	95% confidence interval		ASMR	95% confidence interval		ASMR	95% confidence interval		ASMR	95% confidence interval		ASMR	95% confidence interval	
			Lower	Upper		Lower	Upper		Lower	Upper		Lower	Upper		Lower	Upper		Lower	Upper
Total	1983	56.4	55.5	57.3	69.3	66.7	71.8	22.0	21.3	22.7	28.0	27.0	28.9	78.9	76.6	81.3	232.3	224.2	240.3
	1984	49.5	48.6	50.3	63.6	61.1	66.1	18.0	17.3	18.6	24.2	23.3	25.1	69.3	67.1	71.4	208.3	200.9	215.8
	1985	43.1	42.3	43.9	49.1	46.8	51.4	13.9	13.4	14.5	22.1	21.2	23.0	63.9	61.8	65.9	184.4	177.5	191.2
	1986	37.6	36.9	38.4	36.2	34.2	38.2	10.8	10.4	11.3	19.7	18.9	20.6	58.0	56.1	59.9	165.8	159.4	172.1
	1987	35.8	35.1	36.5	31.4	29.5	33.3	9.7	9.2	10.1	17.8	17.0	18.5	55.0	53.2	56.9	167.1	160.8	173.3
	1988	33.4	32.7	34.1	23.5	21.9	25.1	7.5	7.1	7.9	15.4	14.7	16.1	49.9	48.2	51.7	176.1	169.8	182.3
	1989	30.5	29.8	31.1	20.5	18.9	22.0	5.8	5.5	6.2	13.3	12.7	14.0	44.5	42.9	46.1	170.6	164.5	176.6
	1990	27.1	26.5	27.7	19.8	18.3	21.3	5.1	4.7	5.4	11.7	11.1	12.3	40.2	38.7	41.7	149.5	144.1	154.9
	1991	24.7	24.2	25.3	12.9	11.7	14.2	3.6	3.3	3.9	10.8	10.2	11.4	38.9	37.5	40.4	139.5	134.4	144.6
	1992	24.9	24.4	25.5	12.9	11.7	14.1	3.7	3.4	4.0	10.1	9.5	10.6	35.1	33.7	36.5	153.7	148.4	158.9
	1993	25.9	25.3	26.5	11.9	10.8	13.1	3.2	2.9	3.5	8.8	8.3	9.3	33.5	32.2	34.8	177.0	171.4	182.6
	1994	25.4	24.9	26.0	13.5	12.3	14.7	2.7	2.4	2.9	8.2	7.7	8.7	31.6	30.3	32.8	178.7	173.2	184.2
	1995	22.6	22.1	23.1	9.1	8.1	10.1	2.0	1.8	2.2	7.7	7.2	8.1	29.3	28.1	30.5	159.2	154.1	164.2
	1996	20.3	19.8	20.8	8.9	8.0	9.9	1.7	1.5	1.9	6.5	6.1	6.9	26.3	25.2	27.4	144.2	139.5	148.8
	1997	21.2	20.7	21.6	9.4	8.4	10.4	2.0	1.8	2.3	5.9	5.5	6.3	25.5	24.4	26.6	157.0	152.3	161.8
	1998	25.3	24.8	25.8	7.5	6.6	8.4	1.7	1.5	1.9	7.0	6.6	7.4	32.0	30.8	33.2	190.6	185.4	195.7
	1999	23.6	23.1	24.1	6.9	6.0	7.8	1.3	1.1	1.5	6.1	5.7	6.5	27.2	26.1	28.2	187.7	182.7	192.7
	2000	27.2	26.6	27.7	6.7	5.8	7.6	1.4	1.2	1.6	5.8	5.4	6.1	27.1	26.0	28.2	232.0	226.5	237.5
	2001	22.3	21.9	22.8	4.6	3.8	5.3	1.3	1.1	1.4	5.2	4.9	5.5	23.2	22.2	24.2	187.0	182.2	191.8
	2002	21.1	20.6	21.5	5.4	4.5	6.2	1.0	0.9	1.2	5.0	4.6	5.3	21.4	20.5	22.3	177.5	172.9	182.1
	2003	19.5	19.1	19.9	3.8	3.1	4.6	0.7	0.6	0.9	4.8	4.4	5.1	20.5	19.6	21.3	164.3	160.1	168.6

	2004	19.6	19.2	20.1	3.4	2.7	4.1	0.8	0.7	1.0	4.5	4.2	4.8	18.7	17.9	19.6	170.9	166.6	175.1
	2005	21.1	20.7	21.5	3.6	2.8	4.3	0.8	0.6	1.0	4.0	3.7	4.3	19.4	18.5	20.2	188.3	184.0	192.7
	2006	20.8	20.4	21.1	4.0	3.2	4.8	0.7	0.5	0.8	3.8	3.5	4.1	18.2	17.4	19.0	188.1	183.8	192.3
	2007	19.7	19.3	20.1	3.6	2.8	4.3	0.6	0.4	0.7	3.5	3.2	3.8	17.4	16.7	18.2	179.3	175.3	183.3
	2008	20.6	20.3	21.0	2.8	2.1	3.5	0.7	0.5	0.8	3.2	2.9	3.5	16.6	15.9	17.3	193.7	189.7	197.8
	2009	22.0	21.6	22.3	4.0	3.2	4.8	0.6	0.5	0.7	3.3	3.1	3.6	15.9	15.2	16.6	210.1	206.0	214.1
	2010	23.7	23.3	24.0	4.1	3.3	4.9	0.5	0.4	0.6	3.0	2.8	3.3	17.0	16.2	17.7	229.5	225.4	233.6
	2011	24.4	24.0	24.8	3.7	2.9	4.5	0.6	0.4	0.7	2.8	2.6	3.1	15.9	15.2	16.6	241.8	237.6	245.9
	2012	26.0	25.6	26.4	2.5	1.8	3.1	0.5	0.4	0.7	2.3	2.1	2.5	15.0	14.3	15.6	266.7	262.5	270.9
	2013	24.6	24.2	25.0	2.5	1.8	3.1	0.3	0.2	0.4	2.2	2.0	2.4	14.1	13.4	14.7	253.7	249.7	257.7
	2014	25.1	24.7	25.5	1.6	1.0	2.1	0.4	0.3	0.5	2.0	1.8	2.2	14.5	13.9	15.1	260.0	256.1	263.9
	2015	27.6	27.2	28.0	1.8	1.2	2.3	0.4	0.3	0.5	2.1	1.9	2.3	14.3	13.7	14.9	290.2	286.1	294.2
	2016	28.9	28.5	29.3	2.9	2.1	3.6	0.5	0.4	0.7	1.9	1.7	2.1	15.1	14.5	15.7	303.2	299.2	307.1
	2017	30.1	29.8	30.5	1.9	1.3	2.4	0.4	0.2	0.5	1.6	1.4	1.8	14.0	13.4	14.5	323.3	319.3	327.2
Men	1983	78.6	76.8	80.5	67.2	63.7	70.7	21.9	20.9	22.9	34.6	33.0	36.2	126.7	122.3	131.1	367.1	348.6	385.5
	1984	69.0	67.4	70.7	59.9	56.5	63.3	17.8	16.9	18.7	30.0	28.6	31.4	113.0	109.0	117.1	325.6	309.0	342.2
	1985	61.4	59.8	63.0	50.4	47.2	53.5	13.5	12.8	14.3	28.4	27.0	29.8	103.3	99.4	107.1	290.4	275.0	305.7
	1986	55.1	53.6	56.6	34.2	31.5	36.8	10.6	10.0	11.3	24.7	23.4	26.0	95.5	91.8	99.1	273.4	258.9	288.0
	1987	52.3	50.9	53.7	30.0	27.5	32.6	9.0	8.4	9.6	21.8	20.6	23.0	90.2	86.8	93.7	273.5	259.2	287.8
	1988	50.2	48.8	51.6	22.2	19.9	24.4	7.3	6.7	7.8	19.6	18.5	20.7	83.3	80.0	86.6	286.9	272.7	301.1
	1989	46.1	44.7	47.4	18.7	16.7	20.8	6.0	5.5	6.5	17.0	16.0	18.0	74.2	71.2	77.3	276.9	263.1	290.6
	1990	40.7	39.5	41.9	20.7	18.5	22.8	4.6	4.2	5.1	15.4	14.5	16.4	65.3	62.5	68.1	241.5	229.5	253.5
	1991	37.5	36.4	38.7	12.0	10.4	13.6	3.8	3.3	4.2	14.5	13.6	15.4	63.3	60.5	66.0	224.3	213.0	235.7
	1992	38.6	37.4	39.7	12.5	10.9	14.2	3.8	3.3	4.2	13.9	13.0	14.8	56.6	54.1	59.1	254.4	242.5	266.4
	1993	39.7	38.5	40.9	11.7	10.1	13.2	3.3	2.9	3.7	11.5	10.7	12.3	55.1	52.6	57.5	283.3	270.8	295.8
	1994	39.0	37.9	40.2	14.6	12.9	16.4	2.5	2.1	2.8	11.1	10.3	11.8	51.2	48.9	53.5	286.2	273.9	298.4
	1995	35.2	34.1	36.2	9.7	8.3	11.1	1.8	1.5	2.1	10.6	9.9	11.4	47.0	44.8	49.1	259.3	248.0	270.6
	1996	31.8	30.8	32.8	9.5	8.1	10.9	1.6	1.3	1.8	8.9	8.3	9.6	42.8	40.8	44.9	235.5	225.1	246.0
	1997	33.2	32.2	34.2	9.6	8.1	11.0	1.8	1.5	2.1	8.2	7.6	8.8	41.0	39.0	42.9	258.9	248.3	269.6
	1998	39.4	38.3	40.4	8.0	6.7	9.3	1.7	1.4	2.0	9.7	9.1	10.4	51.6	49.4	53.7	305.0	293.5	316.5
	1999	37.4	36.4	38.4	6.7	5.4	7.9	1.4	1.1	1.6	8.7	8.1	9.3	43.8	41.8	45.7	306.6	295.2	317.9
	2000	41.7	40.6	42.8	7.2	5.9	8.5	1.5	1.2	1.8	8.0	7.4	8.6	42.8	40.9	44.7	361.8	349.5	374.0

	2001	34.6	33.6	35.5	4.4	3.4	5.4	1.4	1.2	1.7	7.5	6.9	8.0	36.3	34.5	38.0	296.1	285.4	306.7
	2002	32.0	31.1	32.9	4.7	3.6	5.8	1.1	0.8	1.3	7.1	6.6	7.6	33.2	31.6	34.9	275.0	264.9	285.0
	2003	29.8	28.9	30.6	4.4	3.2	5.5	0.8	0.6	1.0	6.7	6.1	7.2	31.4	29.8	33.0	255.5	246.1	264.9
	2004	29.8	29.0	30.7	3.9	2.8	4.9	0.9	0.7	1.2	6.5	6.0	7.1	28.9	27.4	30.4	262.2	253.0	271.5
	2005	30.5	29.7	31.4	3.6	2.5	4.6	0.7	0.5	0.9	5.7	5.2	6.2	29.3	27.9	30.8	273.8	264.7	282.9
	2006	29.9	29.1	30.7	3.9	2.7	5.0	0.7	0.5	0.9	5.4	4.9	5.9	27.4	26.0	28.8	271.2	262.4	280.1
	2007	28.5	27.7	29.2	3.6	2.5	4.7	0.7	0.5	0.9	5.3	4.8	5.7	25.8	24.4	27.1	258.5	250.1	266.9
	2008	29.4	28.7	30.2	3.0	2.1	4.0	0.7	0.5	0.9	4.7	4.2	5.1	24.5	23.2	25.7	276.4	267.9	284.8
	2009	30.5	29.8	31.3	4.5	3.3	5.8	0.6	0.4	0.8	4.8	4.4	5.3	24.3	23.1	25.6	288.3	279.9	296.6
	2010	33.6	32.8	34.3	4.2	3.0	5.4	0.6	0.4	0.8	4.3	3.8	4.7	25.9	24.6	27.1	323.5	314.9	332.1
	2011	33.9	33.2	34.7	3.7	2.6	4.8	0.7	0.5	0.9	3.9	3.5	4.3	24.5	23.3	25.7	332.4	323.9	340.8
	2012	36.3	35.6	37.1	2.3	1.4	3.1	0.5	0.3	0.7	3.3	3.0	3.7	23.0	21.8	24.1	369.9	361.3	378.5
	2013	34.1	33.4	34.8	3.0	2.0	4.0	0.4	0.2	0.5	3.2	2.8	3.6	21.8	20.7	22.8	345.6	337.6	353.7
	2014	35.0	34.3	35.7	1.9	1.1	2.7	0.4	0.2	0.5	2.6	2.3	2.9	22.0	20.9	23.0	360.4	352.4	368.3
	2015	38.7	38.0	39.4	2.0	1.2	2.8	0.5	0.3	0.6	2.7	2.4	3.1	22.0	20.9	23.1	403.6	395.5	411.8
	2016	40.1	39.4	40.8	2.8	1.8	3.7	0.5	0.3	0.6	2.5	2.1	2.8	23.2	22.1	24.3	418.1	410.2	426.0
	2017	41.1	40.4	41.8	1.8	1.0	2.6	0.3	0.2	0.5	2.2	1.9	2.6	22.0	20.9	23.0	435.9	428.1	443.7
Women	1983	40.5	39.5	41.5	71.5	67.8	75.2	22.0	21.0	23.0	20.9	19.6	22.1	37.3	35.1	39.6	160.6	152.3	168.9
	1984	35.2	34.3	36.1	67.6	63.9	71.3	18.1	17.2	19.0	18.0	16.8	19.1	31.1	29.1	33.1	143.5	135.9	151.2
	1985	29.6	28.8	30.5	47.8	44.6	51.0	14.3	13.5	15.2	15.5	14.4	16.5	29.5	27.6	31.4	125.7	118.7	132.7
	1986	25.1	24.3	25.9	38.4	35.5	41.4	11.1	10.3	11.8	14.4	13.4	15.4	25.2	23.5	26.9	107.8	101.5	114.1
	1987	24.1	23.3	24.8	32.8	30.0	35.6	10.4	9.6	11.1	13.4	12.5	14.4	24.3	22.6	25.9	109.6	103.3	115.8
	1988	21.5	20.8	22.2	25.0	22.5	27.5	7.7	7.1	8.3	10.8	10.0	11.7	20.5	19.0	22.0	114.9	108.7	121.2
	1989	19.6	18.9	20.2	22.4	20.1	24.8	5.7	5.2	6.2	9.4	8.6	10.2	18.3	16.8	19.7	113.1	107.0	119.2
	1990	17.5	16.9	18.1	18.9	16.7	21.1	5.5	5.0	6.0	7.8	7.1	8.5	18.0	16.6	19.4	98.6	93.2	104.0
	1991	15.5	15.0	16.1	14.0	12.1	15.9	3.5	3.1	3.9	6.9	6.3	7.6	17.5	16.1	18.8	92.6	87.4	97.7
	1992	15.6	15.0	16.1	13.3	11.5	15.1	3.6	3.2	4.1	6.0	5.4	6.6	16.0	14.7	17.2	100.1	94.8	105.3
	1993	16.6	16.0	17.2	12.2	10.5	13.9	3.0	2.6	3.4	6.0	5.4	6.5	14.1	12.9	15.2	120.8	115.2	126.5
	1994	16.2	15.6	16.8	12.2	10.5	13.9	2.9	2.5	3.2	5.2	4.7	5.7	13.6	12.5	14.7	120.8	115.3	126.3
	1995	14.1	13.5	14.6	8.5	7.1	9.9	2.2	1.9	2.6	4.5	4.1	5.0	13.0	11.9	14.0	105.4	100.3	110.4
	1996	12.5	12.0	13.0	8.3	6.9	9.7	1.8	1.5	2.1	4.0	3.5	4.5	10.8	9.8	11.8	95.7	91.0	100.4
	1997	13.2	12.7	13.7	9.2	7.7	10.7	2.2	1.9	2.6	3.4	3.0	3.8	11.0	10.0	12.0	102.7	98.0	107.4

1998	15.8	15.3	16.4	6.9	5.6	8.2	1.7	1.4	2.1	4.1	3.7	4.6	13.5	12.4	14.6	131.0	125.8	136.3
1999	14.6	14.1	15.1	7.2	5.8	8.5	1.3	1.0	1.5	3.4	3.0	3.8	11.4	10.4	12.3	126.0	121.0	131.0
2000	18.0	17.5	18.6	6.2	4.9	7.4	1.3	1.1	1.6	3.4	3.0	3.8	12.0	11.0	13.0	166.0	160.3	171.7
2001	14.4	14.0	14.9	4.8	3.7	5.9	1.1	0.8	1.3	2.9	2.5	3.2	10.6	9.7	11.5	130.3	125.3	135.2
2002	14.1	13.7	14.6	6.2	4.8	7.5	1.0	0.7	1.2	2.8	2.4	3.1	10.0	9.1	10.9	127.7	122.9	132.4
2003	12.9	12.4	13.3	3.3	2.3	4.2	0.7	0.5	0.9	2.8	2.4	3.2	9.7	8.9	10.6	117.0	112.6	121.4
2004	13.0	12.6	13.5	2.8	1.9	3.7	0.7	0.5	1.0	2.4	2.1	2.7	8.8	8.0	9.6	123.0	118.6	127.4
2005	14.9	14.4	15.3	3.6	2.5	4.7	0.9	0.7	1.1	2.2	1.9	2.6	9.6	8.8	10.4	142.3	137.7	146.9
2006	14.8	14.4	15.2	4.2	3.0	5.4	0.7	0.5	0.8	2.1	1.8	2.4	9.3	8.5	10.1	143.4	138.8	147.9
2007	14.1	13.6	14.5	3.5	2.4	4.6	0.4	0.3	0.6	1.6	1.4	1.9	9.2	8.4	10.0	137.4	133.1	141.7
2008	15.0	14.6	15.4	2.6	1.6	3.5	0.6	0.4	0.8	1.7	1.4	1.9	8.8	8.0	9.6	149.8	145.5	154.2
2009	16.3	15.9	16.7	3.5	2.4	4.6	0.6	0.4	0.8	1.8	1.5	2.1	7.6	6.9	8.3	167.1	162.6	171.5
2010	17.3	16.9	17.7	4.0	2.8	5.1	0.4	0.2	0.5	1.8	1.5	2.0	8.1	7.4	8.8	179.1	174.6	183.6
2011	18.3	17.8	18.7	3.7	2.6	4.8	0.5	0.3	0.6	1.7	1.4	1.9	7.3	6.6	7.9	192.6	188.0	197.1
2012	19.4	19.0	19.9	2.7	1.8	3.7	0.6	0.4	0.8	1.2	1.0	1.5	7.0	6.4	7.7	209.7	205.1	214.3
2013	18.4	18.0	18.8	1.9	1.1	2.7	0.3	0.1	0.4	1.1	0.8	1.3	6.5	5.9	7.0	201.7	197.3	206.0
2014	18.8	18.4	19.2	1.2	0.6	1.8	0.4	0.2	0.5	1.3	1.1	1.6	7.2	6.6	7.8	203.4	199.1	207.6
2015	20.6	20.2	21.0	1.6	0.8	2.3	0.4	0.2	0.5	1.4	1.2	1.7	6.7	6.1	7.3	226.0	221.6	230.4
2016	21.6	21.2	22.0	2.9	1.9	4.0	0.6	0.4	0.8	1.3	1.0	1.6	7.0	6.4	7.6	236.0	231.6	240.3
2017	22.8	22.4	23.1	1.9	1.1	2.8	0.4	0.2	0.5	0.9	0.7	1.1	6.1	5.6	6.6	255.1	250.7	259.5

ASMR, Age-standardized mortality rate from infectious diseases (number of infectious disease deaths per 100,000 population)

Supplementary Table S3 Rate ratios based on intrinsic estimator coefficients for age, period and cohort for infectious disease deaths in South Korea from 1983 to 2017

Factor	Total			Men			Women		
	Rate ratio	95% confidence interval		Rate ratio	95% confidence interval		Rate ratio	95% confidence interval	
		Lower	Upper		Lower	Upper		Lower	Upper
Age									
0-4	2.84	2.45	3.29	2.28	1.92	2.70	3.75	3.33	4.23
5-9	0.60	0.51	0.71	0.46	0.38	0.57	0.84	0.74	0.96
10-14	0.26	0.22	0.32	0.22	0.18	0.27	0.34	0.29	0.40
15-19	0.27	0.23	0.32	0.24	0.20	0.29	0.33	0.28	0.38
20-24	0.18	0.16	0.22	0.13	0.11	0.16	0.28	0.25	0.32
25-29	0.20	0.17	0.23	0.17	0.14	0.20	0.27	0.24	0.31
30-34	0.24	0.21	0.28	0.23	0.20	0.27	0.27	0.24	0.30
35-39	0.33	0.29	0.37	0.35	0.31	0.40	0.28	0.25	0.31
40-44	0.45	0.41	0.50	0.53	0.48	0.59	0.31	0.28	0.34
45-49	0.60	0.55	0.66	0.73	0.67	0.80	0.37	0.34	0.41
50-54	0.78	0.72	0.85	0.96	0.88	1.04	0.49	0.45	0.54
55-59	1.05	0.97	1.14	1.28	1.18	1.39	0.70	0.64	0.76
60-64	1.58	1.46	1.70	1.88	1.73	2.04	1.17	1.08	1.26
65-69	2.72	2.51	2.94	3.14	2.89	3.41	2.30	2.14	2.47
70-74	5.46	5.04	5.91	6.08	5.57	6.64	5.34	4.99	5.72
75-79	12.39	11.37	13.50	13.28	12.05	14.63	14.08	13.11	15.13
80-	44.68	40.75	48.98	43.23	38.90	48.04	62.50	57.90	67.48
Period									
1983-1987	2.76	2.61	2.91	2.60	2.45	2.76	3.05	2.91	3.20
1988-1992	1.51	1.43	1.61	1.51	1.42	1.60	1.54	1.46	1.62
1993-1997	1.01	0.96	1.07	1.03	0.97	1.10	0.99	0.94	1.05
1998-2002	0.86	0.81	0.90	0.89	0.84	0.93	0.83	0.80	0.87
2003-2007	0.61	0.58	0.64	0.62	0.59	0.66	0.60	0.57	0.63
2008-2012	0.63	0.60	0.67	0.63	0.59	0.67	0.63	0.60	0.66
2013-2017	0.71	0.67	0.76	0.71	0.67	0.76	0.68	0.65	0.72
Cohort									
1903	0.31	0.26	0.37	0.38	0.30	0.48	0.25	0.22	0.28
1908	0.52	0.45	0.60	0.62	0.52	0.73	0.44	0.39	0.49
1913	0.92	0.82	1.02	1.09	0.96	1.23	0.77	0.70	0.84
1918	1.50	1.38	1.64	1.75	1.58	1.93	1.26	1.17	1.36
1923	2.20	2.04	2.38	2.45	2.25	2.67	1.91	1.79	2.04
1928	2.76	2.58	2.95	2.91	2.70	3.14	2.56	2.42	2.70
1933	3.19	3.00	3.38	3.20	2.99	3.42	3.10	2.95	3.26
1938	3.18	2.95	3.42	3.11	2.87	3.37	3.13	2.92	3.35
1943	3.24	2.96	3.54	3.05	2.77	3.35	3.43	3.15	3.73
1948	2.95	2.65	3.28	2.70	2.41	3.02	3.28	2.96	3.64
1953	2.91	2.59	3.28	2.73	2.41	3.10	3.03	2.70	3.40
1958	2.75	2.42	3.12	2.63	2.29	3.01	2.73	2.41	3.08

1963	2.46	2.13	2.84	2.37	2.03	2.77	2.41	2.11	2.75
1968	1.98	1.69	2.33	1.88	1.58	2.24	2.05	1.78	2.36
1973	1.59	1.34	1.90	1.50	1.24	1.83	1.67	1.44	1.94
1978	1.23	1.03	1.47	1.15	0.94	1.40	1.33	1.15	1.53
1983	0.68	0.58	0.79	0.64	0.54	0.77	0.72	0.64	0.81
1988	0.45	0.36	0.55	0.40	0.31	0.51	0.52	0.44	0.61
1993	0.37	0.29	0.47	0.35	0.27	0.47	0.40	0.33	0.48
1998	0.25	0.18	0.34	0.22	0.15	0.32	0.29	0.22	0.37
2003	0.19	0.12	0.30	0.19	0.11	0.31	0.21	0.14	0.30
2008	0.18	0.11	0.29	0.17	0.10	0.30	0.19	0.13	0.29
2013	0.11	0.05	0.22	0.11	0.05	0.24	0.11	0.06	0.21
